# *ABCC6* and Pseudoxanthoma Elasticum: The Face of a Rare Disease from Genetics to Advocacy

**DOI:** 10.3390/ijms18071488

**Published:** 2017-07-11

**Authors:** Karobi Moitra, Sonia Garcia, Michelle Jaldin, Clementine Etoundi, Donna Cooper, Anna Roland, Patrice Dixon, Sandra Reyes, Sevilay Turan, Sharon Terry, Michael Dean

**Affiliations:** 1 Department of Biology, Trinity Washington University, College Of Arts and Sciences, 125 Michigan Avenue NE, Washington, DC 20017, USA; GarciaS@students.trinitydc.edu (S.G.); JaldinM@students.trinitydc.edu (M.J.); EtoundiC@students.trinitydc.edu (C.E.); CooperD@students.trinitydc.edu (D.C.); RolandA@students.trinitydc.edu (A.R.); DixonP@students.trinitydc.edu (P.D.); ReyesS@students.trinitydc.edu (S.R.); TuranS@trinitydc.edu (S.T.); 2 PXE International, 4301 Connecticut Avenue NW, Suite 404, Washington, DC 20008, USA; sterry@pxe.org; 3 Laboratory of Translational Genomics, The Division of Cancer Epidemiology and Genetics (DCEG), National Cancer Institute, National Institutes of Health, Gaithersburg, MD 20885, USA

**Keywords:** pseudoxanthoma elasticum, mineralization, rare disease, next generation sequencing, patient advocacy

## Abstract

Pseudoxanthoma elasticum (PXE) is an autosomal recessive disorder characterized by the mineralization of connective tissues in the body. Primary manifestation of PXE occurs in the tissues of the skin, eyes, and cardiovascular system. PXE is primarily caused by mutations in the *ABCC6* gene. The *ABCC6* gene encodes the trans-membrane protein ABCC6, which is highly expressed in the kidneys and liver. PXE has high phenotypic variability, which may possibly be affected by several modifier genes. Disease advocacy organizations have had a pivotal role in bringing rare disease research to the forefront and in helping to sustain research funding for rare genetic diseases in order to help find a treatment for these diseases, pseudoxanthoma elasticum included. Because of these initiatives, individuals affected by these conditions benefit by being scientifically informed about their condition, having an effective support mechanism, and also by contributing to scientific research efforts and banking of biological samples. This rapid progress would not have been possible without the aid of disease advocacy organizations such as PXE International.

## 1. Introduction

Pseudoxanthoma elasticum (PXE) is a genetic disorder that affects multiple bodily systems. It is estimated to affect between 1 in 25,000 and 1 in 100,000 people. The distinguishing feature of PXE is mineralization of the soft connective tissue in primarily the skin, eyes, and the arterial blood vessels [1]. The skin lesions of a person affected by PXE usually consist of small, yellowish bumps. The bumps or papules usually join together to form large patches and most commonly appear on the side of the neck and progress to other flexor areas, such as the antecubital fossa (inside the elbow), groin, and popliteal fossa. The eye lesions affect most patients with PXE between the ages 20 and 40, although early eye signs are very common [1,2,3,4,5]. This rare disease occurs in all ethnicities. There is currently no known effective treatment for PXE overall, however, signs like eye bleeds can be somewhat effectively managed using the current treatments for age-related macular degeneration. PXE is caused primarily by mutations in the *ABCC6* gene. This gene encodes the transmembrane protein ABCC6, which may function as a transporter and is mainly expressed in the kidneys and liver [1,2,3,4,5]. PXE is a disease with high phenotypic variability and more than 300 mutations have been documented in the *ABCC6* gene [1]. 

## 2. Discovery and Structure of the *ABCC6* Gene

**Figure 1:**
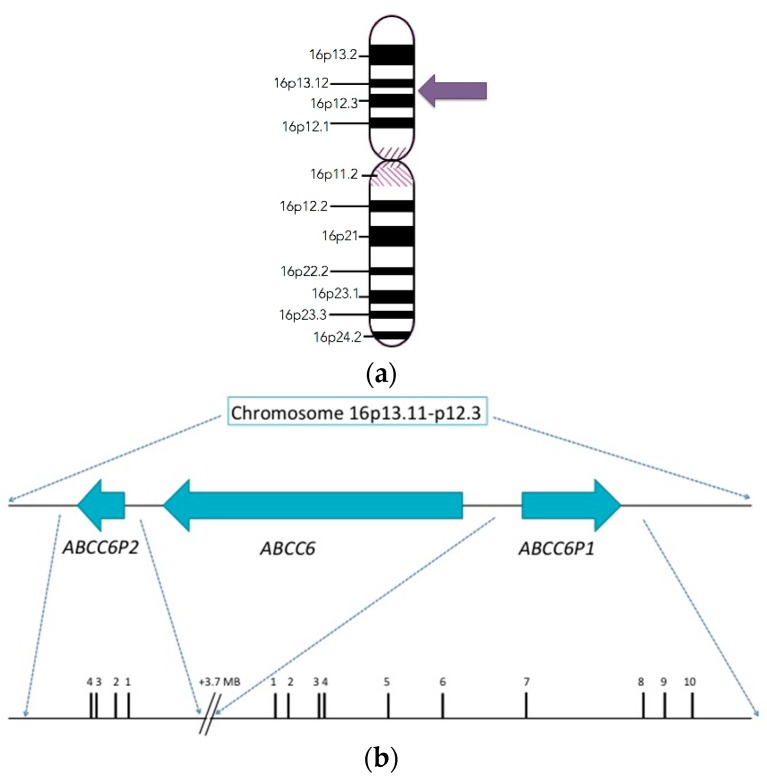
(**a**) The Chromosomal location of the *ABCC6* gene. The location of the *ABCC6* gene is on chromosome 16 at the specific locus 16p13.1 it spans approximately 75 kb of DNA and is composed of 31 exons; (**b**) Cartoon representation of the genomic organization of the 2 transcribed pseudogenes of *ABCC6-ABCC6P1* and *ABCC6P2*. The pseudogenes flank the parental *ABCC6* gene on chromosome 16p13. Arrows indicate the direction of transcription. The exons of *ABCC6P1* and *ABCC6P2* genes are shown in more detail. Exons are represented by black vertical lines and numbered in 5′ to 3′ order. All the pseudogene exons share 98–100% identity with the parental *ABCC6* except exon 10 of *ABCC6P1* which shares 18.7% identity and is non-homologous.

Since the pathologic effects of PXE are observed to affect the elastic fibers in skin, the genes involved in the synthesis and assembly of elastic fiber network were originally considered as the primary candidates for mutations. Some of the former candidates included elastin (ELN), elastin associated microfibrillar proteins (FBN1 and FBN2), and lysyl oxidase (LOX) [1]. The gene thought to be responsible for PXE was discovered at the turn of the century in the year 2000 by PXE International and members of the PXE International Research Consortium (PIRC). The main players in the discovery were Sharon Terry (who holds the gene patent) and Drs. Jouni Uitto and Arthur Bergen. They localized the gene to the short arm of chromosome 16. The gene was previously designated as the *MRP6* gene in reference to the MRP6 protein, but the proper designation is the *ABCC6* gene. The specific location of this gene is 16p13.1 (Figure 1) and it encompasses approximately 75 kb of DNA and is comprised of 31 exons. The mRNA encodes a polypeptide of 1503 amino acids. There are also two pseudogenes related to the *ABCC6* gene upstream of it in the genome. ABCC6-Ψ1 is the first pseudogene, this encompasses the upstream gene region and the homologous sequences ranging from exon 1 through intron 9. *ABCC6Ψ2*, is the second pseudogene this gene includes the upstream nucleotide sequence and the nucleotides between exon 1 and intron 4. [1,4,6,7]. There is 99% sequence similarity between *ABCC6* and its pseudogenes. The *ABCC6* gene is primarily expressed in the liver, proximal tubules of the kidney, and at a very low level (if at all) in PXE affected tissues of patients [1,4,6,7].

## 3. Structure of the ABCC6 Protein and Its Functional Role in PXE

**Figure 2:**
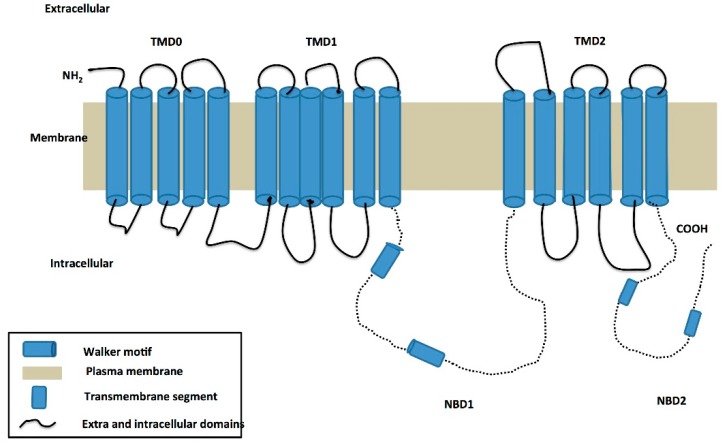
The predicted two-dimensional topology of the ABCC6 protein. The ABCC6 protein is predicted to contain three transmembrane domains (TMDs)—TMD0, TMD1, and TMD2. TMD0 is predicted to be comprised of five TM alpha helices, while the other two TMDs contain six TM helices apiece, giving a total of 17 TM helices making up the full transporter. The dotted lines represent the Nucleotide Binding Domains (NBD’s) and *ABCC6* has 2 of them. The Walker motifs in the NBD’s are represented by the blue boxes between the dotted lines. The plasma membrane is represented in brown, while the TMD’s are represented by blue cylinders and the extracellular and intracellular domains (loops) are represented by unbroken lines.

The ABCC6 protein contains three transmembrane domains (TMDs)—TMD0, TMD1, and TMD2. TMD0 is predicted to consist of five transmembrane alpha helices, while the other TMDs contain six, giving a total of 17 transmembrane helices (Figure 2).

Certain residues in the transmembrane domains of ABC transporters, usually in TMD1 and TMD2, have been postulated to be the sites for binding of selective substrates and also may form a translocation path for substrates [8,9]. The role of TMD0 is unclear in ABCC6. The nucleotide binding domain (NBD) organization is similar to the typical organization of most ABC transporters, and both NBDs in ABCC6 contain a Walker A, Walker B, C-loop, and other conserved motifs indicative of an ATPase domain of an ABC transporter. The nucleotide binding domain of transporters couples ATP hydrolysis to substrate transport. The NBD’s bind and hydrolyze ATP providing the energy for substrate transport. The NBD’s typically contain highly conserved motifs such as a Walker A site that contains a phosphate binding loop, Walker B which contains the magnesium binding site, the histidine loop which is considered to be the switch region, the signature sequence LSGGQ (C-loop) which is specific to ABC transporters and the Q-loop which is present between the Walker A and C-loop, its function is to interact with gamma phosphate [8,9,10].

### Predicted Functional Role of ABCC6 in PXE

Sequence homology studies with other members of the C sub-family of ABC proteins (such as ABCC1), have suggested that ABCC6 functions as an efflux transporter in the liver. In vitro studies investigating the role of the ABCC6 transporter demonstrated that it may function in the transport of glutathione-conjugated molecules. The role of the *ABCC6* gene in the pathogenesis of PXE has also been validated through the study of the corresponding mouse gene *ABCC6*, which is similar to the human *ABCC6* gene. In mouse knock-outs for *ABCC6*, the mouse phenotype was progressive tissue mineralization [1]. 

## 4. Founder Effect in PXE

A founder effect is said to occur when a new population is established by a few founding members of an original population. If, as a result of the founder effect, the newly established population becomes insulated within its members then there is reduced genetic variation in the resulting population. The founder effect has been observed in several human populations. The Afrikaner population seems to have a high prevalence of PXE that can be explained by the existence of a founder effect in this population. It has been reported in the black population in the Cape Province of South Africa that the prevalence of PXE is 1/650,000, however, a much higher prevalence of 1/23,000 was observed in the Afrikaner community of the Cape Province. The Afrikaner population of the Cape Province is of Dutch, German, and French descent. These populations settled in the Cape of Good Hope during the 17th century. The basis for the high prevalence of PXE in the Afrikaner population may be the result of a founder effect in this population [11,12]. From mutational analysis and haplotype data from 24 families, it was established that the European-derived Afrikaner population that had settled in South Africa in the 17th century carried three common haplotypes and six different disease-causing variants. Haplotype analysis of these families revealed that the three most frequent mutations—R1339C, Y768X, and R1138Q are identical-by-descent, which suggests a founder origin of PXE in this population [13]. 

## 5. Clinical Manifestation of PXE

Clinical manifestations in PXE patients include primarily skin, ocular, and cardiovascular manifestations in patients.

### 5.1. Skin Phenotype

**Figure 3:**
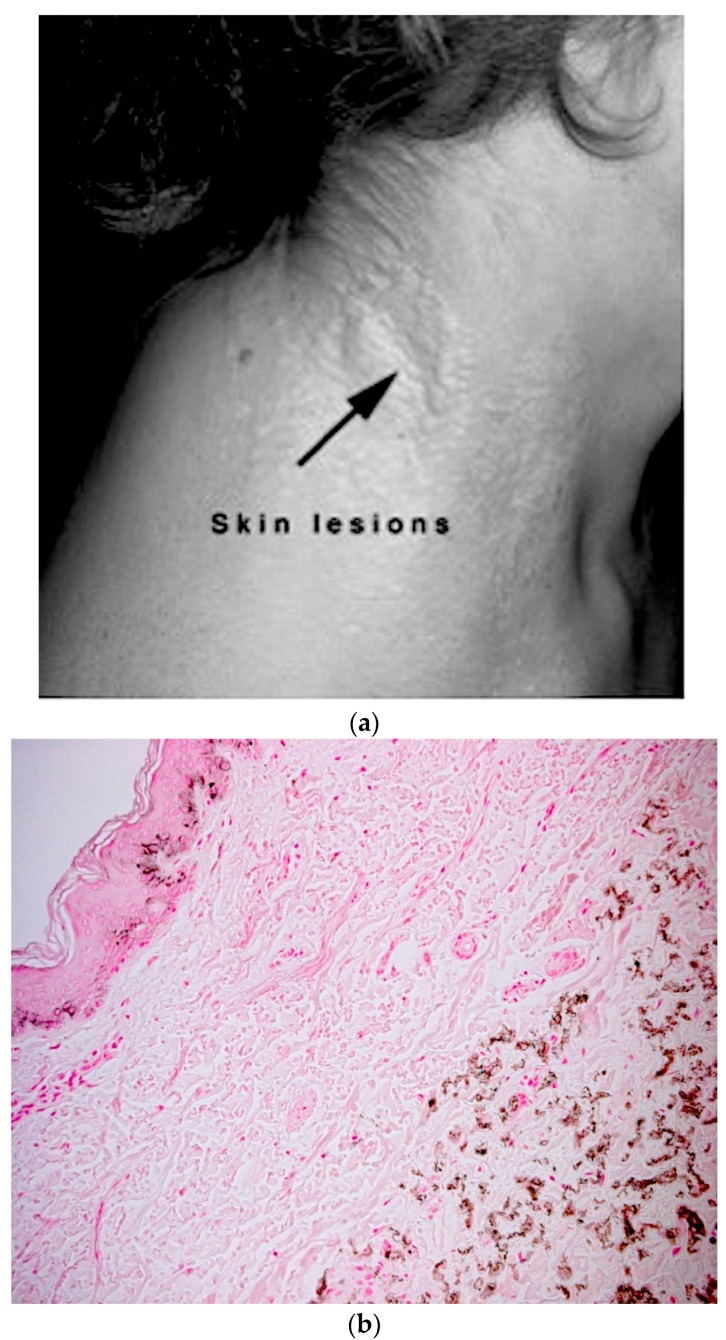
(**a**) Papules on the neck of a pseudoxanthoma elasticum (PXE) patient. Calcification of the elastic fibers in the skin may lead to cutaneous lesions, which are small, yellowish, flat papules that develop typically on the neck, as depicted in the photograph; (**b**) Skin biopsy of a PXE patient depicting calcium deposition. The tissue has been stained with von Kossa staining, showing the calcium deposition in the tissue (brownish-red granules). Photograph is the property of PXE International. It has been used with permission and collected with approval from Genetic Alliance IRB #00003999, PXE001 protocol.

PXE causes visible changes to the skin of the PXE patient that vary from person to person. The calcification of the elastic tissue in the skin leads to cutaneous lesions, which are small, yellowish, flat papules, resembling a rash which develops typically on the neck (Figure 3a). These may coalesce into leathery plaques and cause the skin to become wrinkly and loose [1,6]. The small, yellowish, flat papules are also observed in other flexural areas of the body such as the neck, the underarms, the skin on the inside of the elbows, the groin, and the skin behind the knees in PXE patients [1,6]. As the disease progresses, the affected skin becomes lax and wrinkled due to loss of elasticity, resulting in skin-folds. Lesions may also appear on mucous membranes such as inside of the lower lips or lining of the rectum or vagina [6]. The histological characteristics of skin lesions can be identified through a skin biopsy and subsequent von Kossa staining, which would show calcium deposition in the tissue (Figure 3b). 

### 5.2. Ocular Manifestations

**Figure 4:**
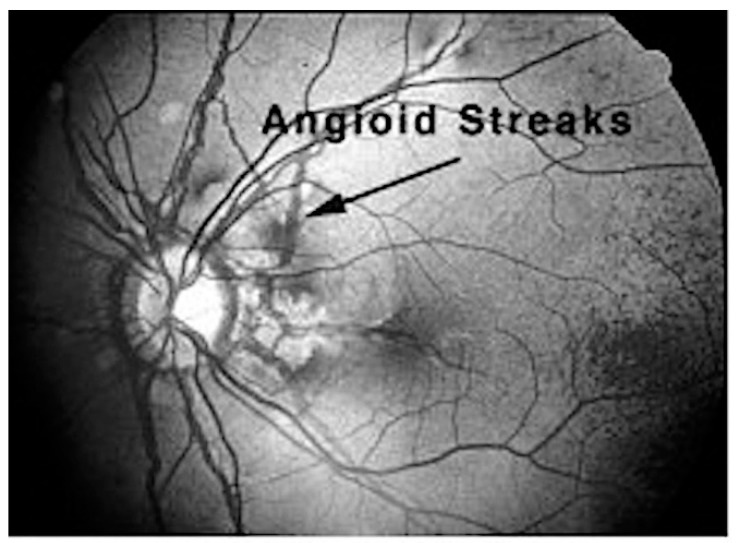
Ocular manifestation of PXE in the form of angioid streaks in the eye of a PXE patient. Angioid streaks are reddish brown or grey lines that may radiate from the optic disc (arrow) and result from calcification of elastic fibers of the retina. Angioid streaks may lead to cracks in the elastic membrane (Bruch’s membrane) behind the retina.

Ocular manifestations occur due to the loss of elasticity within the extracellular matrix between the retinal pigment epithelium and the choroid [14]. One criterion for the ophthalmologic diagnosis of PXE is ‘peau de orange’ or mottling of the retina that may progress into angioid streaks. Angioid streaks are irregular, reddish brown or grey lines that radiate from the optic disc that result from degeneration and calcification of elastic fibers of the retina (Figure 4). Angioid streaks may lead to cracks in the elastic membrane behind the retina (Bruch’s membrane) due to enhanced calcification of the membrane [15]. These cracks in the Bruch’s membrane may cause new blood vessels to grow from the choroid into the retina and result in the progressive loss of central vision [1]. Absent treatment, individuals with ocular manifestations of PXE may lose much of their central vision and can eventually become legally blind even though they continue to have peripheral vision [6]. Recently, anti-angiogenesis drugs injected into the retina in the area of an active bleed appear to limit its impact and generally vision is salvaged, albeit diminished in some cases.

### 5.3. Cardiovascular Manifestations

Dutch PXE patients may have increased risk of cardiovascular disease. Studies suggest that sporadic claudication (pain due to poor circulation of blood in the arteries) is one of the first signs of cardiovascular symptoms in PXE patients [2]. PXE can cause mineralization of the mid-laminar layers of arteries, which causes narrowing of the blood vessels [6]. Vascular manifestations of PXE are due to degeneration of elastic lamina of medium-sized arteries and calcium deposition [15]. This narrowing may cause individuals to have decreased blood flow to the arms and legs, cause a heart attack, small strokes, hypertension (high blood pressure) intermittent claudication, occasional bleeding from the gastrointestinal vessels, and high cholesterol. 

### 5.4. Diagnosis of PXE

Histological characteristics of skin lesions are elastic tissue and calcium deposition identified by von Kossa staining. The major criteria used for the diagnosis of PXE is either a positive von Kossa stain and family history of PXE, or positive von Kossa stain and ophthalmologic features such as peau d’orange and angioid streaks [15,16]. The minor criteria for diagnosis include histological features of non-lesional skin and also family history of PXE in first-degree relatives. Genetic testing may be used to confirm diagnosis of PXE.

## 6. Therapeutic Intervention in PXE

Currently there is no systemic therapy that mitigates or slows the progression of PXE. Restrictions of calcium intake, increase in vitamin K intake, and magnesium as a therapy have all been tried but have failed. The signs and symptoms of PXE can be treated by various methods, such as surgery to treat loose or sagging skin [1] and anti-angiogenic drugs to treat the abnormal growth of retinal blood vessels [6]. 

## 7. The Role of ABCC6 in PXE

In the literature, there are three hypotheses postulating the role of ABCC6 in PXE: the Metabolic Hypothesis, the PXE Cell Hypothesis, and, most currently, the Extracellular ATP Release Hypothesis.

### 7.1. The Metabolic Hypothesis

The metabolic theory puts forward the hypothesis that a lack of ABCC6 functional activity (mainly in the liver) results in the deficiency of certain circulatory factors that are required to prevent abnormal mineralization in the peripheral tissues. There is some support for this theory in studies involving the *ABCC6* knock-out mouse model [7] and from various in vitro studies [17]. ABCC6’s function is to prevent mineralization but under standard calcium and phosphate non-homeostatic conditions can cause mineralization if mutations are present [1]. It was observed that PXE patients and *ABCC6*^−/−^ (knock-out) mice that have similar PXE outcomes [1]. Serum from both PXE patients and the *ABCC6^−/−^* mice lacked the ability to prevent calcium and phosphate deposition. Furthermore, it was shown that when serum from patients with PXE was added to tissue culture medium it altered the expression of elastin by fibroblasts (elastin enables skin to stretch). This demonstrates that there is both a presence or an absence of certain circulatory factors in PXE patients that play a crucial role in determining mineralization in PXE patients [1]. 

### 7.2. The PXE Cell Hypothesis

This hypothesis postulates that the absence of ABCC6 expression in affected tissues changes their biosynthetic expression profile and cell to extracellular matrix interactions which gives rise to alterations in the proliferative capacity of cells. One line of evidence in support of this theory is that in cultured skin fibroblasts from PXE patients, MMP-2 (Matrix-Metalloprotease-2) activity is enhanced, which may lead to an enhanced potential for degradation of these cells [18]. 

### 7.3. The Extracellular ATP Release Hypothesis

It was recently discovered that the factor which may prevent mineralization in PXE patients is pyrophosphate, which can act as a mineralization inhibitor [19]. It was suggested, based on studies conducted in vitro with cultured HEK293 liver cells, that ABCC6 acts as a regulator for the mediation of extracellular ATP release from the cell. This extracellular ATP is hydrolyzed into AMP and pyrophosphate by the ectonucleotidases. It was also shown later with the aid of cultured primary hepatocytes [20] that ABCC6 by some unknown mechanism may mediate the direct sinusoidal release of ATP from hepatic cells. Thus, the extracellular ATP release hypothesis indicates that ABCC6 acts as a regulator in the process of cellular ATP release.

## 8. Common Mutations and Variations in *ABCC6* Associated with PXE

The four most frequent PXE mutations in Caucasians are *R1141X*, exon 23–29 deletion, *R1164X*, and *Q378X*. R1141X is in exon 24 and accounts for about 30% of all PXE mutations. The second most common alteration is AluI-mediated deletions of exons 23 to 29 (del 23–29)—this has been found in at least 20% of PXE patients in the United States (on at least one allele). Other recurrent mutations are: nonsense mutations (a codon is changed to a stop codon) Q378X in exon 9, R518X in exon 12, R1164X in exon 24, and a clustering of nonsense mutations in exons 24 and 28 which are present in the NBDs that are critical for ABCC6 function. Analysis of missense mutations indicated that frequencies of the mutations residing in the domain-domain interfaces are 3.5–4.1-fold higher than those over the entire protein.

## 9. Next Generation Sequencing Approaches for Detecting Variants in *ABCC6* and Modifier Genes

Next generation sequencing approaches may be one way to detect variants in *ABCC6* and modifier genes in the laboratory.

### 9.1. 454 Sequencing

Roche-454 technology is the first next-generation sequencing technology that utilizes a DNA amplification method known as emulsion PCR (EmPCR). During emPCR streptavidin beads carrying DNA fragments can be captured into specific emulsion droplets to create copies of a unique DNA template per bead. These droplets serve to amplify the reaction and produce around 10^7^ clonal copies of a unique DNA template/bead [21].

**Figure 5:**
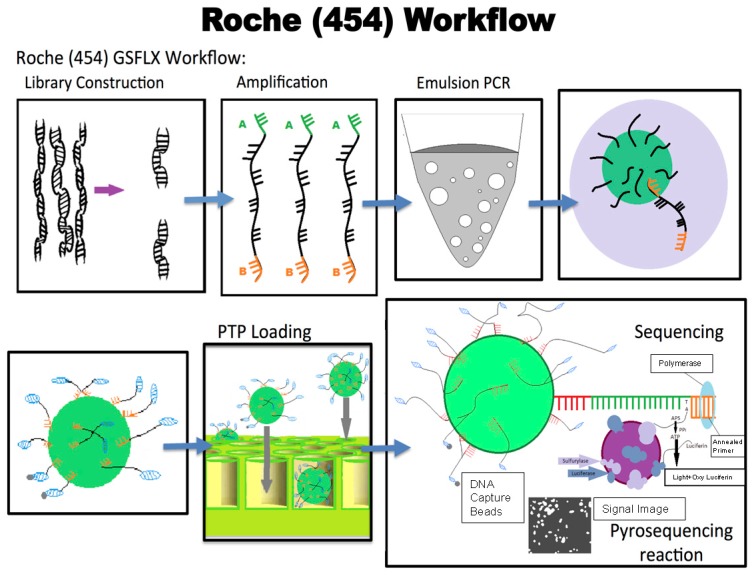
Overview of the Roche-454 technology for next-generation sequencing. The Roche-454 technique allows thousands of pyrosequencing reactions to be carried out in parallel. The DNA templates are sequenced using a sequencing-by-synthesis approach. As each nucleotide is added it is detected through a chemiluminescent reaction and documented photographically. In detail: when a complimentary nucleotide is added to the template strand it releases a pyrophosphate that ultimately produces ATP through enzyme catalyzed reactions. The enzyme sulfurylase assists in the overall biochemical reaction that helps convert pyrophosphate (PPi) to ATP when adenosine 5′ phosphosulfate (APS) is present in the reaction. Luciferase can then oxidize luciferin in the presence of this ATP to form oxyluciferin in a reaction that produces a light signal each time a nucleotide is added. This light is documented by a camera and can be visualized in a pyrogram. The blue arrows depict the flow of the process from library construction through to sequencing. The purple arrows show the actual pyrosequencing process that is described in the text. The light purple arrow in the right hand corner box depicts sulfurylase and adjacent blue arrow depicts luciferase. The vertical arrows in right hand corner box depict the generation of ATP and the light emitting reaction. PTP: Picotiter Plate.

Each template-containing bead is then transferred into a well of a picotiter plate for sequencing. This technique allows thousands of pyrosequencing reactions to be carried out in parallel. The templates are then sequenced using a “sequencing by synthesis” approach through which the complimentary strand is synthesized and as each nucleotide is added it is detected and documented (Figure 5) [21]. Light is produced by a chemiluminescent enzyme for each nucleotide that is added. This is captured by a camera to document the bases being incorporated into the DNA strand. In 454 Technology, the chemiluminescent signal intensity is proportional to the amount of pyrophosphate released and the number of bases incorporated [21]. 

### 9.2. Ion Torrent Sequencing

In this technology, nucleotides are detected as they are incorporated into the nascent strand. As each nucleotide is added it releases a hydrogen ion that is detected by sensors. Initially the DNA to be sequenced is fragmented then ligated to adapters. Subsequently, the adaptor-ligated libraries are clonally amplified onto beads. The sequencing primers and DNA polymerase are then added. The reaction mixture is pipetted into the chip’s loading port for sequencing. As sequencing progresses, each nucleotide is incorporated into the nascent strand by the bound polymerase. This increases the length of the sequencing primer usually by one base/nucleotide. Incorporation of a nucleotide results in the hydrolysis of the incoming nucleotide triphosphate, which causes the release of a single proton for each nucleotide incorporated. A shift in pH is produced due to the release of the proton. Proton release is detected by the sensor on the bottom of each well, converted to a voltage, and finally digitized by electronics. Basically, each well acts as a mini pH meter to detect changes in hydrogen ion concentration. After the flow of each nucleotide, the wells are washed to make sure that nucleotides do not remain in the well [22].

## 10. Modifier Genes and PXE

A number of modifier genes have been linked to PXE. In the context of PXE, we define modifier genes as genes with the capacity to alter the effect that another gene produces. Modifier genes may help explain the variation and degrees of symptoms (phenotypic variability) that individuals with PXE experience.

By searching the literature, we discovered 13 modifier genes with a variety of functions, including antioxidant and cellular stress genes such as catalase and *SOD2*. Genes involved in mineralization and calcium metabolism pathways, such as *XYLT1/2*, *MGP*, and *MMP2*, genes involved in the breakdown of ATP, such as *ENPP1*, and genes involved in angiogenesis, such as *VEGFA*. Another modifier gene, *SPP1*, may also be associated with PXE. Polymorphisms in the promoter of the *SPP1* gene is a genetic factor that may correlate with PXE [1]. Screening of promoter mutations revealed three of nine different sequence variations were significantly more frequent in PXE patients than in controls. It may be hypothesized that polymorphisms in the *SPP1* promoter are a genetic risk factor that contributes to PXE [23].

## 11. The Role of Disease Advocacy Groups in PXE and Other Rare Genetic Disorders

In the United States, a disease is rare when less than 1 in 200,000 are affected by it, but the definition of a rare disease varies by country [24].

### 11.1. The PXE Story

In 1881, Rigal, the French dermatologist, first described PXE [22]. In 1896, Darier differentiated between common xanthomas and PXE [25]. However, in the years following the discovery of this disease there was little research on PXE. As a result, physicians were ill-informed about this rare genetic disorder, leading to contradictory opinions in the medical profession, resulting in confusion and distress to affected individuals. This dismal scenario underwent a sea change with the birth of disease advocacy groups for rare diseases in the early 1990s. One of these advocacy groups was PXE International founded by Sharon and Patrick Terry, the parents of two children affected with PXE.

### 11.2. The Birth of PXE International—For the People, of the People, by the People

There was a high demand for support, information, and research infrastructure for pseudoxanthoma elasticum (PXE) [26]. This was very clear to the founders of PXE International (the advocacy organization for PXE), Patrick and Sharon Terry, when their children were diagnosed with PXE in 1994 [27]. Her children provided blood to two different research laboratories in a short time because both groups were competing against each other and did not share information with one another [27]. The Terrys saw this competition as unjust and ineffective for patients, physicians, and researchers alike. To improve this ineffective system, they founded PXE International in 1995 [6]. PXE International is a small non-profit organization that promotes research and support for individuals affected by PXE. It began and continues to a large extent to be funded through private donations, which over the years has also included small and sporadic grants from foundations, companies, and the federal government. Their mission is to initiate, fund, and conduct research, provide support for individuals and families affected by PXE and provide resources for healthcare professionals [6]. PXE international conducts basic and clinical research and provides financial support for applied translational research, product and treatment development, and works on behalf of individuals and their families to improve the quality of life through advancing research and education [6]. It has also achieved success in accelerating translational research in PXE, and has been used as a model for other advocacy organizations [26].

### 11.3. The Role of Patient Advocates in the Discovery of the ABCC6 Gene

In 1995, the Terrys initiated a project to find the gene responsible for the disease with the help of friends, neighbors, fellow patient advocates, and researchers/physicians in the field [28]. First, PXE International founded a blood and tissue bank to aggregate enough samples to do positional cloning. They worked in a lab at night and learned to clone genes. They created and led a research consortium to accelerate research on PXE [26]. In 2000, PXE International and collaborating researchers discovered that mutations in the gene *ABCC6* were the primary cause of PXE [26]. Sharon Terry filed for a patent on the gene as a co-discoverer, along with researchers from the University of Hawaii. She assigned her rights to PXE International and it acts as a “steward” for the gene and to represent the interests of the PXE community by moving from gene discovery to diagnostics [26]. This patent stands to protect all of the patients affected with PXE and to promote further research involving the *ABCC6* gene. 

### 11.4. Other Genetic Disorders and the Role of Genetic Alliance

Genetic Alliance is a leading nonprofit health advocacy organization committed to engaging individuals in transforming health. Genetic Alliance’s mission is to engage individuals, families, and communities in transforming health [29]. They support individuals and families that are living with genetic conditions and disorders. Genetic Alliance is a dynamic network that includes more than 1200 disease-specific advocacy organizations promoting an open space for shared resources, creative tools, and innovative programs [26]. Since 1995, Genetic Alliance has established research agendas and worked with medical specialty groups to accelerate these agendas, and to collect clinical information and biological samples [30]. They are supported by funding from government contracts and grants, industry and corporate support, fees generated by services and events, and by individual donations [29]. Genetic Alliance and the Terrys believe that all stakeholders, including affected individuals, families, clinicians, and researchers, should work together to advance research and services on a given condition. They designed a map, called Navigating the Ecosystem of Translational Science, to aid collaboration and facilitate coordinated and sequential activities. As a part of this overarching plan, they believed that stakeholders needed a common infrastructure beginning with a biorepository and quickly moving to registries to promote further research to determine the genetic basis of diseases [27].

### 11.5. The Genetic Alliance Registry and BioBank (GARB)

As a scaling up and expansion of the PXE International Biobank, the Genetic Alliance BioBank was founded in 2003 as a centralized repository of biological samples and data from across many diseases to enable translational genomic research on rare genetic diseases and to breakdown silos [26]. GARB provides disease organizations the necessary tools to manage collections [30]. All data has formal consent along with clinical and environmental information [26]. Member organizations can retain donor identifiers and distribute coded samples to ensure confidentiality. All members of GARB comply with human participant regulations, and with the US Health Insurance Portability and Accountability Act (HIPAA) [26]. This repository is a new model where the need and rights of all parties are acknowledged and protected [26]. This Biobank also provides access to all components that are necessary, such as access to its own IRB (Institutional Review Board) for approval to collect and archive DNA and tissue samples [26]. The system also includes a web-based user interface that complies with the regulations of the US food and Drug Administration (FDA) [26]. The infrastructure of GARB allows advocacy organizations a low-cost effective mechanism to pursue sophisticated, novel research collaborations to promote sufficient research in diseases [26]. This is vital for advocacy organizations because this allows them to perform and promote further research in genetic diseases [29]. Being part of Genetic Alliance allows researchers to procure biological material to further their research studies. The very existence of GARB means that researchers are not forced to chase patients around the globe to receive biological samples for their studies or to try to use samples in siloed collections [30]. GARB was a huge step forward for not only researchers, but also individuals with certain diseases. 

Another important part of GARB is the Platform for Engaging Everyone Responsibly (PEER), a registry platform that enables individuals to indicate their preferences about data sharing, access, and privacy. PEER allows cross condition aggregation of information managed by the individuals. It collects patient reported outcomes using validated instruments and disease specific surveys. There are more than 40 sponsors ranging from disease advocacy organizations to professional societies and community health groups. More than 20,000 people have enrolled in the system. Genetic Alliance’s purpose is to make this a resource available to different organizations in order to share infrastructure, costs, platform, messaging, and shared standards across all disease groups [29].

## 12. Conclusions

It has been established that PXE is a rare hereditary disease and that people affected by PXE typically have a normal life span. However, maintaining a healthy life style is vital for a better quality of life [15]. Through the efforts of many researchers the database of PXE mutations has grown throughout the years. Some of these mutations have clarified the genetic basis of PXE. It has been found that most *ABCC6* gene mutations are clustered in the NBD region of the protein, towards the carboxyl-terminal end [31]. The clustering of mutations present at the NBD of the ABCC6/MRP6 proteins indicates that these regions are vital for maximal function of the protein. Although there has been much progress regarding studies on variations in the *ABCC6* gene and also in PXE modifier genes, further studies are essential to determine the specific molecules being transported by the ABCC6 protein [32,33]. Advocacy organizations have paved the way towards a greater role for patients in managing their disease and also in contributing towards research efforts. The Genetic Alliance Registry and Biobank has served as a model for disease research and collaboration. One advocacy organization, PXE International, has helped other patient advocacy groups create their own registries and biorepositories, which serve as catalysts to continue further studies for finding treatments and cures for many diseases. Bringing organizations together accelerates research efforts and assists researchers in gaining access to biological samples and associated clinical information. This enables clinicians, researchers, policy makers, and affected people to come together and collaborate toward a common goal: to find the cure.

## References

[1] Qiaoli L., Qiujie J., Pfendner E., Váradi A., Uitto J. (2009). Pseudoxanthoma elasticum: Clinical phenotypes, molecular genetics and putative pathomechanisms. Exp. Dermatol..

[2] Bergen A.A., Plomp A.S., Schuurman E.J., Terry S., Breuning M., Dauwerse H., Swart J., Kool M., van Soest S., Baas F. (2000). Mutations in ABCC6 cause pseudoxanthoma elasticum. Nat. Genet..

[3] Le Saux O., Urban Z., Tschuch C., Csiszar K., Bacchelli B., Quaqlino D., Pasquali-Ronchetti I., Pope F.M., Richards A., Terry S. (2000). Mutations in a gene encoding an ABC transporter cause pseudoxanthoma elasticum. Nat. Genet..

[4] Pfendner E.G., Vanakker O.M., Terry S.F., Vourthis S., McAndrew P.E., McClain M.R., Fratta S., Marais A.S., Hariri S., Coucke P.J. (2007). Mutation detection in the ABCC6 gene and genotype-phenotype analysis in a large international case series affected by pseudoxanthoma elasticum. J. Med. Genet..

[5] Ringpfeil F., Pulkkinen L., Uitto J. (2001). Molecular genetics of pseudoxanthoma elasticum. Exp. Dermatol..

[6] PXE International. WWW.PXE.org.

[7] Klement J.F., Matsuzaki Y., Jiang Q.J., Terlizzi J., Choi H.Y., Fujimoto N., Li K., Pulkkinen L., Birk D.E., Sundberg J.P. (2005). Targeted ablation of the *ABCC6* gene results in ectopic mineralization of connective tissues. Mol. Cell. Biol..

[8] Borths E.L., Locher K.P., Lee A.T., Rees D.C. (2002). The structure of Escherichia coli BtuF and binding to its cognate ATP binding cassette transporter. Proc. Natl. Acad. Sci. USA.

[9] Higgins C.F. (2001). ABC transporters: Physiology, structure and mechanism—An overview. Res. Microbiol..

[10] Schneider E., Hunke S. (1998). ATP-binding-cassette (ABC) transport systems: Functional and structural aspects of the ATP-hydrolyzing subunits/domains. FEMS Microbiol. Rev..

[11] Miksch S., Lumsden A., Guenther U.P., Foernzler D., Christen-Zäch S., Daugherty C., Ramesar R.K., Lebwohl M., Hohl D., Neldner K.H. (2005). Molecular genetics of pseudoxanthoma elasticum: Type and frequency of mutations in ABCC6. Hum. Mutat..

[12] Torrington M., Viljoen D.L. (1991). Founder effect in 20 Afrikaner kindreds with pseudoxanthoma elasticum. S. Afr. Med. J..

[13] Le Saux O., Beck K., Sachsinger C., Treiber C., Göring H.H., Curry K., Johnson E.W., Bercovitch L., Marais A.S., Terry S.F. (2002). Evidence for a founder effect for pseudoxanthoma elasticum in the Afrikaner population of South Africa. Hum. Genet..

[14] Chassaing N., Martin L., Calvas P., Bert M.L., Hovnanian A. (2005). Pseudoxanthoma Elasticum: A Clinical, Pathophysiological And Genetic Update Including 11 Novel ABCC6 Mutations. J. Med. Genet..

[15] Laube S., Moss C. (2005). Pseudoxanthoma elasticum. Arch. Dis. Child..

[16] Lebwohl M., Phelps R.G., Yannuzzi L., Chang S., Schwartz I., Fuchs W. (1987). Diagnosis of pseudoxanthoma elasticum by scar biopsy in patients without characteristic skin lesions. N. Engl. J. Med..

[17] Le Saux O., Bunda S., Van Wart C.M., Douet V., Got L., Martin L., Hinek A. (2006). Serum factors from pseudoxanthoma elasticum patients alter elastic fiber formation in vitro. J. Investig. Dermatol..

[18] Quaglino D., Sartor L., Garbisa S., Boraldi F., Croce A., Passi A., De Luca G., Tiozzo R., Pasquali-Ronchetti I. (2005). Dermal fibroblasts from pseudoxanthoma elasticum patients have raised MMP-2 degradative potential. BBA Mol. Basis Dis..

[19] Jansen R.S., Küçükosmanoğlu A., de Haas M., Sapthu S., Otero J.A., Hegman I.E., Bergen A.A., Gorgels T.G., Borst P., van de Wetering K. (2013). ABCC6 prevents ectopic mineralization seen in pseudoxanthoma elasticum by inducing cellular nucleotide release. Proc. Natl. Acad. Sci. USA.

[20] Jansen R.S., Duijst S., Mahakena S., Sommer D., Szeri F., Váradi A., Plomp A., Bergen A.A., Elferink R.P., Borst P. (2014). ATP-Binding Cassette Subfamily C Member 6-Mediated ATP Secretion by the Liver Is the Main Source of the Mineralization Inhibitor Inorganic Pyrophosphate in the Systemic Circulation. Arterioscler. Thromb. Vasc. Biol..

[21] Morozova O., Marra M.A. (2008). Applications of next-generation sequencing technologies in functional genomics. Genomics.

[22] Rothberg J.M., Hinz W., Rearick T.M., Schultz J., Mileski W., Davey M., Leamon J.H., Johnson K., Milgrew M.J., Edwards M. (2011). An integrated semiconductor device enabling non-optical genome sequencing. Nature.

[23] Hendig D., Arndt M., Szliska C., Kleesiek K., Götting C. (2007). SPP1 promoter polymorphisms: Identification of the first modifier gene for pseudoxanthoma elasticum. Clin. Chem..

[24] Uitto J. (2012). Rare heritable skin diseases: Targets for regenerative medicine. J. Investig. Dermatol..

[25] Pseudoxanthoma Elasticum. Pseudoxanthoma Elasticum.

[26] Terry S.F., Terry P.F., Rauen K.A., Uitto J., Bercovitch L.G. (2007). Advocacy groups as research organizations: The PXE International example. Nat. Rev. Genet..

[27] Landy D.C., Brinich M.A., Colten M.E., Horn E.J., Terry S.F., Sharp R.R. (2012). How disease advocacy organizations participate in clinical research: A survey of genetic organizations. Genet. Med..

[28] Allen A. Who Owns My Disease?. http://www.motherjones.com/politics/2001/11/who-owns-my-disease.

[29] Genetic Alliance. www.geneticalliance.org.

[30] Levenson D. (2011). Genetic alliance marks 25 years. Am. J. Med. Genet. Part A.

[31] Moitra K. (2012). ABC transporters in human disease. Colloquium Series on The Genetic Basis of Human Disease.

[32] Uitto J., Váradi A., Bercovitch L., Terry P.F., Terry S.F. (2013). Pseudoxanthoma elasticum: Progress in research toward treatment: Summary of the 2012 PXE International Research Meeting. J. Investig. Dermatol..

[33] Uitto J., Li Q., van de Wetering K., Váradi A., Terry S.F. (2017). Insights into Pathomechanisms and Treatment Development in Heritable Ectopic Mineralization Disorders: Summary of the PXE International Biennial Research Symposium-2016. J. Investig. Dermatol..

